# Variability in the Histopathological Diagnosis of Non-Melanocytic Lesions Excised to Exclude Melanoma

**DOI:** 10.5826/dpc.1104a94

**Published:** 2021-10-01

**Authors:** Ian Katz, Tony Azzi, Alister Lilleyman, Blake O’Brien, Brian Schapiro, Curtis Thompson, Tarl Prow

**Affiliations:** 1 Southern Sun Pathology, Thornleigh, NSW, & School of Medicine, University of Queensland, QLD, Australia; 2 Newcastle Skin Check, Charlestown, NSW, & School of Medicine, University of Queensland, QLD, Australia; 3 Sullivan Nicolaides Pathology, Bowen Hills, & School of Medicine, University of Queensland, QLD, Australia; 4 CTA Pathology, Oregon, USA; 5 Oregon Health and Sciences University, Oregon, USA; 6 Future Industries Institute, University of South Australia, SA, Australia; 7 Skin Research Centre, York Biomedical Research Institute, Hull York Medical School, University of York, York, United Kingdom

**Keywords:** melanoma, diagnosis, seborrheic keratosis, artificial intelligence, AI, large cell acanthoma

## Abstract

**Introduction:**

The differential diagnosis of lesions excised to exclude melanoma include a variety of benign and malignant melanocytic and non-melanocytic lesions.

**Objectives:**

We examined the variability between pathologists in diagnosing non-melanocytic lesions.

**Methods:**

As part of a larger study prospectively examining the diagnosis of lesions excised to exclude melanoma in 198 patients at a primary care skin cancer clinic in Newcastle, Australia, we compared diagnosis made by 5 experienced dermatopathologists, of 44 non-melanocytic lesions in 44 patients aged 22–90.

**Results:**

Forty-four lesions (out of 217 in total) were non-melanocytic. Among the 5 pathologists who examined each case there was marked variability in the terminology used to diagnose each case. The most common variability was found between seborrheic keratosis, large cell acanthoma, solar lentigo, and lichenoid keratosis. The diagnosis made by the majority of the pathologists was deemed to be the reference diagnosis. Versus majority diagnosis, 4% of benign lesions were considered malignant, and 7% of malignant diagnoses were considered as benign.

**Conclusions:**

The different terminology adopted and lack of consensus in the diagnosis of these non-melanocytic lesions in this setting suggests that training AI systems using gold standards may be problematic. We propose a new management classification scheme called MOLEM (Management of Lesions Excised to exclude Melanoma) which expands the previously described MPATH-dx to include non-melanocytic lesions.

## Introduction

Clinically concerning skin lesions are commonly excised to rule out melanoma [1, 2]. Common clinical mimics of melanoma, excluding the various forms of naevi, include benign and malignant entities such as seborrheic keratosis (SK), solar lentigo (SL), hemangioma, pigmented actinic keratosis, large cell acanthoma (LCA), lichen planus-like keratosis/lichenoid keratosis (LPLK), pigmented intraepithelial squamous cell carcinoma (ISCC), or even pigmented basal cell carcinoma (BCC) [3]. Cysts [4] and dermatofibromata [3] may also be occasionally excised to exclude melanoma. These lesions are generally easily managed. The consequence of misdiagnosis is less serious than for melanoma. However, these lesions represent a significant workload in dermatopathology.

The difficulty in diagnosing melanocytic lesions, especially borderline lesions, among pathologists, and how using a management-based response such as the melanocytic pathology assessment tool and hierarchy for diagnosis (MPATH-Dx) can improve the classification of such lesions has been reported [5]. There is significant overlap in the understanding of many of the benign lesions excised to exclude melanoma, particularly in sun-damaged skin, with many clinicians and pathologists struggling to define and distinguish lesions such as SK, LCA, SL and LPLK [6–10]. Similarly, with regard to malignant lesions that can be included in the clinical differential diagnosis of melanoma, a 2008 report from Ramos-Ceballos et al described only moderate pathologists’ agreement (0.575 concordance) in a curated series of actinic keratosis and squamous cell carcinoma in situ cases [11]. This supports the hypothesis that there is a considerable difficulty when diagnosing both benign and malignant non-melanocytic lesions that are in the differential diagnosis of melanoma. With the recent advances made in whole slide imaging technology it may soon be possible to use artificial intelligence (AI) to support the diagnosis. AI will however require a gold standard diagnosis [12].

In this report, we present the first prospective study evaluating the agreement between dermatopathologists on the diagnosis of non-melanocytic lesions to exclude melanoma. Forty-four non-melanocytic lesions out of 217 total lesions, including melanocytic lesions, were biopsied to exclude melanoma. Biopsies were collected in a prospective manner to exclude case-selection bias. Thus, this case set represents a collection of non-melanocytic clinical mimics of melanoma. Five pathologists diagnosed each case, results show a significant variation in the diagnostic terminology, which aligns with prior reports on melanocytic lesion diagnostic terminology [5]. Variability was highest with SK, LCA, and SL diagnoses. We used a consensus diagnosis for comparison. A total of 4% of benign lesions were over-diagnosed and 7% of malignant lesions were underdiagnosed. The highest levels of agreement were found when diagnosing BCC, cysts, DF, and angioma. The remaining 37 cases had varying terminology regarding actual diagnosis. These results support the need to develop a classification schema that clarifies diagnoses by eliminating the natural language currently in use for diagnosis. This will help to bridge the gap between human pattern recognition and automated diagnosis.

## Methods

### Human Ethics and Volunteers

Cases in this report were collected as part of a larger prospective study on the preclinical, clinical, and histological diagnosis of lesions excised to exclude melanoma, with ethics approval by Bellberry Human Research Ethics Committee, Australia (protocol ID 2018-08-613-A-3). Patients were recruited prospectively from April to December 2019 in a primary care skin cancer clinic in Newcastle, NSW, Australia. Patients with a suspicious lesion that required shave or formal excision to exclude melanoma were asked to participate in the study. All non-melanocytic lesions including a single case thought by only one pathologist to be melanocytic in origin, and lentigo simplex (LS) were included in this report.

#### Pathologist Diagnoses

The first author of this article (IK) reviewed each case and chose one or more representative sections including relevant immunohistochemistry stains. Five experienced dermatopathologists from Australia and the USA reviewed each case. Each pathologist had more than 8 years’ experience and each examined, on average, more than 15,000 skin samples/year. The Australian pathologists reviewed the glass slides while the 2 USA pathologists reviewed digital slides. There were no significant differences between glass slide and digital diagnoses. Pathologists were informed about the patients’ age, sex, and basic clinical details, present on the pathology request form. They were then asked to (1) provide a one-line diagnosis, and (2) assign a risk class according to what we term MOLEM (Management of Lesions to Exclude Melanoma) (Table 1). The MOLEM schema was adapted from the MPATH-Dx classification [5]. The slides presented for each case were taken as representative of the lesion including the assumption that the lesion went to the margins of the excision. The lesion diagnoses fell within MOLEM classes I (benign) and V (malignant). The majority MOLEM class diagnosis was taken as reference standard in terms of benign versus malignant. If there was a tie in the majority MOLEM diagnostic class, the malignant class was assumed to be the gold standard.

#### Slide Scanning and Raw Data

Slide scanning with a Leica AT-2 scanner (magnification × 40) and digital slides were uploaded to Pathpresenter digital slide presentation platform for those pathologists who were assigned to review the digital slides.

#### Statistical Analysis

Statistical analysis was conducted using Microsoft Excel 2016 and GraphPad Prism v7.03.

## Results

A total of 217 lesions from 198 patients were biopsied to exclude melanoma. Within that biopsy pool 44 lesions from 44 patients were non-melanocytic. The average age of the patients was 67 years, there were 20 male and 24 female patients. One case was thought, by just one pathologist, to be melanocytic in origin and was included in this study. We included LS in the non-melanocytic lesions. Details concerning the site of the lesion, patients’ age, and the final diagnosis made by the 5 pathologists for all examined lesions, are listed in Table 2. Diagnoses included SK or a variant of SK, AK, pigmented AK, ISCC, LCA, SL, LS, and LPLK. Cysts, BCC, a dermatofibroma, and an angioma were also included.

There was marked variation in the diagnostic terminology used by the pathologists. Discrepant diagnoses (benign versus malignant) are highlighted in red (Table 2). Pathologists gave 219 interpretations in total (44 lesions × 5 pathologists). One pathologist did not report one case. The 7 cases that were BCC, cysts, DF, and the angioma had no discrepant diagnoses. Of the other 37 cases, 12 (32%) received essentially the same diagnosis from all pathologists but with a slightly different terminology. If we exclude the 7 cases of BCC, cysts, DF, and angioma, 5% of the majority benign diagnoses were considered to be malignant by at least 1 pathologist, and 22% of the majority malignant diagnoses were considered to be benign by at least 1 pathologist (Table 3). If all lesions are included, 4% of the majority benign lesion diagnoses were considered to be malignant, and 7% of the majority malignant diagnoses were considered to be benign (Table 4).

**Figure 1:**
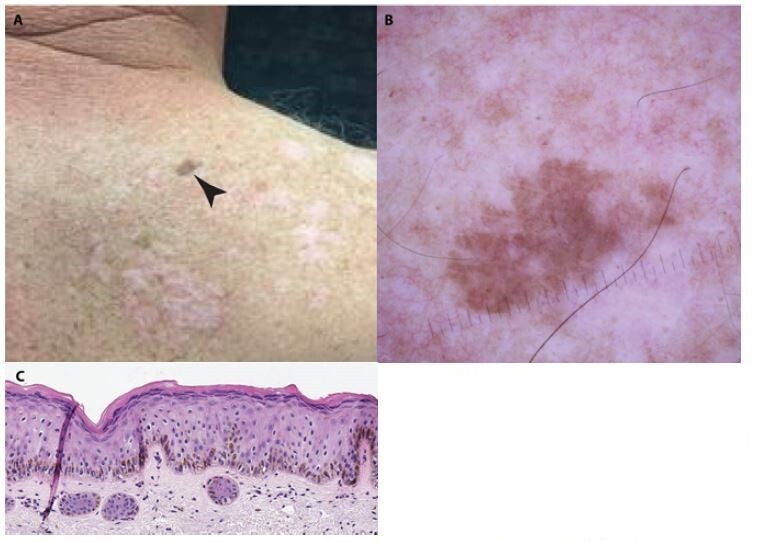
(A) Clinical image showing a solitary asymmetric pigmented macule in sun-damaged skin of upper back. (B) Dermoscopy shows central brown dots and grey areas, and at 6–9 o’clock possible abnormal network with rhomboidal structures, and at 1–3 o’clock features of regression. (C) Stained pathology slide (H&E, ×20), cropped from the Pathpresenter WSI shows slightly thickened epidermis but with minimal cytologic atypia and most pigment in basal keratinocytes. This is a link to the WSI: https://pathpresenter.net/#/public/display?token=34352199.

**Figure 2:**
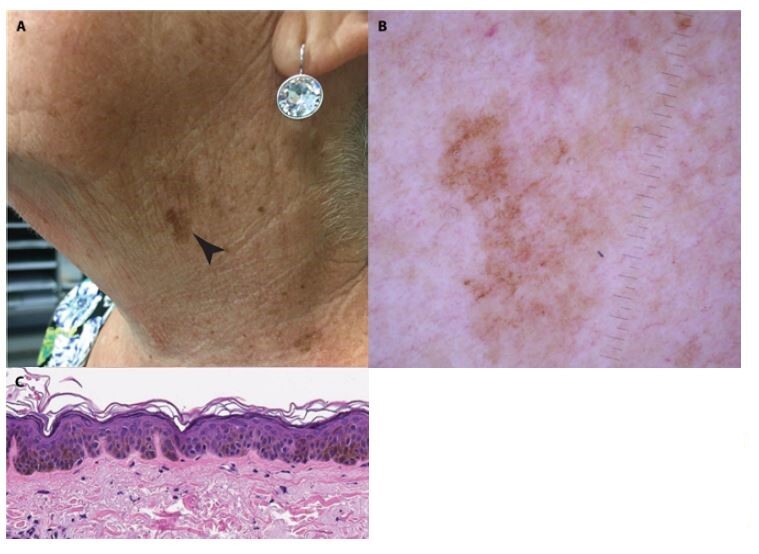
Lesion unanimously diagnosed as solar lentigo. (A) Clinical and (B) dermoscopic images show broad lentiginous pigment with some asymmetry and a possible abnormal network. (C) Pathology (H&E, ×20), showing bulbous acanthosis of epidermis hyperkeratosis increased basal pigmentation, mainly in keratinocytes. The WSI images are found at https://pathpresenter.net/#/public/display?token=1d8609e3.

**Figure 3:**
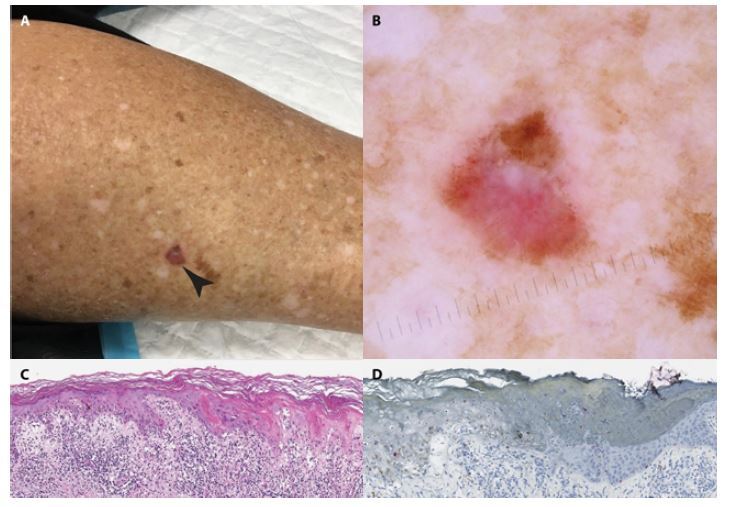
Lesion that was either defined as lichenoid keratosis, intra-epidermal squamous cell carcinoma, or seborrheic keratosis by pathologists participating to this study. (A) Clinical image shows an asymmetrical lesion with red/pink and brown within sun damaged skin. (B) Dermoscopy shows a pink and brown, traumatized lesion, possible peripheral network, and central inflammation. (C) Dense lichenoid chronic inflammatory infiltrate with interface change with some squamous atypia that for some pathologists was diagnostic for intra-epidermal squamous cell (H&E, ×10). (D) SOX-10 immunohistochemistry (magnification 10x) with no significant increase in melanocytes. The WSI image of the H&E can be found at https://pathpresenter.net/#/public/display?token=0c88e847.

Figures 1–3 illustrate 3 representative cases. Figure 1 was taken from a 59-year-old male (Table 2, case 6) with a lesion on the upper back with the clinical history “? MIS”. This represents one of the cases where different diagnoses were given by the 5 pathologists. The suggested diagnoses included pigmented flat SK, LCA, early SK, and SL. Figure 2 was taken from a 73-year-old female presenting with a lesion on the left side of the neck, (Table 2, case 32) with a clinical history of “? melanocytic lesion”. The case shown in Figure 2 is the one where complete diagnostic agreement was found between pathologists. The case was diagnosed as a solar lentigo. Figure 3 shows a 65-year-old woman presenting with a lesion on her calf (Table 2, case 13). Three different diagnoses were made in this case: LPLK with reactive change, early pigmented ISCC, and SK.

## Discussion

This is the first prospective study comparing the diagnosis made by dermatopathologists of non-melanocytic lesions excised to exclude melanoma. Anecdotally, we observed that there was marked variability in the terminology adopted by 5 experienced dermatopathologists to describe and diagnose LPLK, SL, SK, and LCA. This prospective study also reported some disagreement between benign versus malignant lesions’ diagnoses. Benign lesions were over-diagnosed by 4% and malignant lesions were under-diagnosed by 7% compared to majority diagnosis.

Subjectivity in the assessment of melanocytic lesions has been well documented in the literature [5] but our study is the first to prospectively show subjectivity with non-melanocytic lesions. Our results reflect inter-observer variability in a set of otherwise common lesions. There is controversy in both the literature and clinical practice regarding the relationship between LCA, SK, LPLK, SL, and AK [7]. Whether LCA is a distinct entity, or a subtype of SK is still a matter of debate. Sanchez and Requena report that LCA was a distinctive entity [10]. However, Rowert and Ackerman asserted that LCA is a variant of SL, and that SL (including the large cell variant) is a stage in the evolution of reticulated SK and of LPLK [9]. Rhabhari and Pinkus reported that LCA was an AK [8]. On the other hand, Fraga and Amin [6] investigated whether LCA is a variant of solar lentigo by comparing macroscopic, microscopic, and immunophenotypic attributes of LCA with conventional solar lentigo, seborrheic keratosis, actinic keratosis, and Bowen disease. They concluded that LCA is best considered a variant of solar lentigo with cellular hypertrophy. All of this helps explain why there is such low agreement in the diagnoses between these entities among pathologists

Distinguishing a SK from ISCC can occasionally be challenging, both histologically and clinically [13–15]. An accurate diagnosis differentiating benign versus malignant is important. The resulting diagnosis determines treatment options which could severely impact patients with lesions in cosmetically sensitive areas. There are case reports of malignant transformation of SK to SCC and ISCC within a SK is not uncommonly seen in routine dermatopathology practice. There is debate about whether this represents true transformation, chance observation of collision between the two types of lesions, or initial misdiagnosis [13]. There are a number of studies suggesting that immunohistochemical stains could be used to distinguish SK from SCC, eg Ki-67 and p16 [14], or BCL-2 and IMP3 [15], but this is not generally used in routine clinical practice. In the current study, there were 5 lesions (11% of lesions) for which difficulty in distinguishing SK from ISCC was reported.

It can be argued that distinguishing histologically benign lesions has no clinical consequences. However, we argue that making a consistent diagnosis is important for 2 main reasons. Firstly, we observed that the differential diagnosis of keratotic lesions including intra-epithelial squamous cell carcinoma of acanthotic type, and their management could be quite different to the management of morphologically similar benign lesions. Secondly, as AI becomes more prevalent in assisting both clinical and histopathological diagnoses of these lesions, it is vital to establish gold standard diagnoses for training AI systems. We hypothesize that the high level of difficulty in accurately diagnosing the lesions histologically to produce a gold standard diagnosis is likely to significantly reduce AI performance [16]. This can be addressed by extensive research on the subject and through the integration of molecular diagnoses.

Adamson and Welch discussed the problems in deriving a gold standard diagnosis in pathology [12]. Any inherent bias in the data used to train an AI algorithm, will reflect on the final result. The potential for biased data to negatively influence AI-based programs was made evident in the healthcare sphere [17]. Thus, in the early stages of AI in skin pathology, we will have to face the problem of how to create a dataset with minimal bias in terms of disease classification and diagnoses. To help address the issue of variation in the diagnostic terminology we developed a classification schema called MOLEM (Management of Lesions excised to Exclude Melanoma) that groups lesions with similar management strategies. This schema extends upon the MPATH-Dx developed by Piepkorn et al [5], which only accommodates melanocytic lesions.

To create more harmony in the diagnosis of the non-melanocytic lesions excised to exclude melanoma, we suggest several approaches Firstly, the features of each lesion could be better defined in a consensus type meeting, with examples depicted as has been demonstrated in the development of the MPATH-Dx system [5]. Molecular studies may help, as well as correlation with clinical and dermoscopic images, because the clinical impression can often be more typical than the histological impression. An alternative would be to term many of the lesions all “benign keratoses” or “benign keratinocytic lesion” with a note that few potential entities fall under this umbrella, and it is impossible to accurately distinguish them. It is unclear which approach would be most beneficial when defining entities for training AI algorithms. The use of an ‘umbrella’ term may accurately reflect how lesions such as SL, SK, LPLK, and LCA exist on a morphologic spectrum, but over-simplifying classification systems might hinder the potential for machine-based learning algorithms to offer new, and previously unrecognized insights into disease biology. Regarding the issue of distinguishing benign from malignant lesions in 4% to 7% of cases, it would be beneficial to add a note with the lesions’ diagnosis to explain the diagnostic difficulty and recommend a clinical follow-up. On a similar note, it has been suggested that dividing lesions into 3 categories: benign, malignant, and “I do not know”/uncertain” and place a comment with the latter category, might be helpful. It has been suggested that the use of AI for the diagnosis of skin lesions will be “more consistent and replicable than those based on human interpretation, but they may not be any closer to the truth” [12].

There are a few limitations to this study. Firstly, this is a small study with 5 dermatopathologists submitting a diagnosis for each case. Another potential weakness of the study is that some pathologists looked at routine glass slides and other pathologists looked at digital slides. Finally, because these were not routine reports, there is the risk that the slides were not examined in as much detail as normal.

## Conclusions

We show that there is significant inter-pathologist variation in the terminology and diagnosis of benign lesions excised to exclude melanoma. Inter-pathologist variation in distinguishing benign lesions from malignant lesions was also observed. We have proposed a new management classification scheme called MOLEM (Management of Lesions Excised to exclude Melanoma) which expands the previously described MPATH-dx to include non-melanocytic lesions. Our hypothesis is that this approach has the potential to 1) clarify communication between pathologists and clinicians for improved clinical management, and 2) provide a structured diagnostic schema for future work in AI and molecular diagnostics fields.

## Supplementary Material

All pathology slides for the lesions discussed in this paper are available at: https://pathpresenter.net/#/public/presentation/display?token=d08ddedf

## Figures and Tables

**Table 1:** MOLEM Reporting Schema for Skin Lesions’ Classification Into 5 Classes.

MOLEM class	Suggested management	Examples
**I**	No further treatment or topical	Benign naevus, low grade atypia, seborrheic keratosis, lichenoid keratosis, cyst, dermatofibroma, large cell acanthoma
**II**	Narrow but complete excision <5mm	Moderately atypical naevus, Spitz
**III**	Complete excision with > 5mm but < 10mm margins	Melanoma in situ, severely atypical naevus
**IV**	Complete excision with > 10mm margins	Invasive melanoma
**V**	Non-melanoma skin cancer management, possibly including complete excision	Basal cell carcinoma, in situ and invasive squamous cell carcinoma

**Table 2:** Pathologists’ Diagnoses for the 44 Cases.

CASE	P1	P2	P3	P4	P5	MAJORITY CLASS
1	SL	LS	SL	SL	SL	1
2	PAK	PAK	SL AND SK	LCA, AK AND ISCC	ISCC EPIDERMOLYTIC HK	1
3	SL	SL/EARLY SK	SL	SL	SK	1
4	PIG ISCC ARISING IN SK	SK, MACULAR, HEAVILY IGMENTED	SK, INFLAMED	SK	SK	1
5	PIG ISCC, AK	AK WITH ADNEXAL EXTENSION, PIGMENTED	PAK	ISCC	PAK, FOCAL ISCC	5
6	PIG FLAT SK/LCA	SL/EARLY SK	SL/EARLY SK	LCA	SK	1
7	PIG ISCC		PIG ISCC	ISCC	ISCC	5
8	SL WITH LICHENOID REGRESSION	MACULAR SK	SL/EARLY SK, INFLAMED (EARLY LPLK)	SL, LPLK	SK	1
9	SL/FLAT SK WTH LICHENOID REGRESSION	SK, MACULAR, HEAVILY PIGMENTED	SL/EARLY SK, INFLAMED	AK, SL, LPLK	SL/SK	1
10	SL, CHANGES OF REGRESSION, POSSIBLE LPLK	SL	LK	LK	POST INFLAMATORY PIGMENTATION	1
11	SL	SK, MACULAR HEAVILY PIG	SL	SL	SL	1
12	PIG SK	SK	SL, AK	SL, PAK	SK	1
13	PIG EARLY ISCC WITH EROSION, LICHENOID INFLAMATION	LPLK	LPLK	LPLK	SK	1
14	SL	SL	SL, INFLAMED	SL	EPHELIS, SL	1
15	SL	SL	PAK	SL	SK	1
16	SK	SK, PIG	SK	SK	SK	1
17	FAVOUR IRRITATED FLAT SK	LS WITH UNDERLYING STASIS	SL/EARLY SK	LCA	LK	1
18	ISCC ARISING IN SK	SK, PIGMENTED	FAVOUR CLONAL SK OVER ISCC	SK	SK	1
19	PAK, EARLY ISCC	PAK	PAK, INFLAMED	PAK	PAK	1
20	PAK	PAK	PAK	PAK	AK	1
21	PAK AND AL	PAK, HYPERTROPHIC	PAK	PAK	AK	1
22	SK	SK	SL/EARLY SK	SK	SK	1
23	LPLK	LPLK	SK, INFLAMED (EARLY LPLK)	LPLK, POSSIBLE SUBTLE LENTIG PROLIFERATION	LPLK	1
24	IRRITATED SK, DERMAL INFLAMATION	SK INFLAMED	ATYPICAL SQUAMOUS PROLIFERATION, INFLAMED	ISCC	AK	5
25	LATE STAGE LPLK	LICHENOID DERMATITIS WITH LATE STAGE LPLK	LPLK (END STAGE)	LPLK	SL	1
26	SK AND PITYRIASIS VERSICOLOUR	SL AND EARLY SK	SL/SK	LCA	SL	1
27	SK	SK	SK	SK	SK	1
28	POROKERATOSIS	LPLK LATE STAGE	POROKERATOSIS AND SL	LPLK	POROKERATOSIS	1
29	SL	LENTIGO	SL	LCA	SL	1
30	OVERLAPPING SL AND PIG SK	SK, RETICULATED AND PIGMENTED	SK AND SK, INFLAMED	PAK, SL, LCA	SL/SK	1
31	SK	MACULAR SK	AK, SLIGHTLY INFLAMED	SK	SK	1
32	SL	SL	SL	SL	SL	1
33	SL	SL	SL/EARLY SK	SL	SK	1
34	SK	PIG SK	SK, PIGMENTED	SK	SK	1
35	PIG SK	SK	SK, INFLAMED	SK	SK	1
36	SK	SK	SK	SK	SK	1
37	SL	LENTIGO	SL	JMN WITH ATYPIA	SL	1
38	BCC	BCC PINKUS TYPE	BCC	BCC	BCC	5
39	PIG BCC, SUPERFICIAL TYPE	BCC, PIGMENTED	BCC	BCC	BCC	5
40	DF	DF	DF	DF	DF	1
41	PIG BCC	BCC, NODULAR, PIGMENTED	BCC, PIGMENTED	BCC	BCC	5
42	TC	PILAR/TC	FOLLICULAR CYST, ISTHMICTYPE (PILAR OR TRICHILEMMAL)	PC	PC	1
43	BCC	BCC SUPERFICIAL AND NODULAR	BCC NODULAR	BCC	BCC	5
44	FAVOUR LYMPHANGIOMA OVER HEMANGIOMA	LYMPHANGIOMA	BENIGN VASCULAR NEOPLASM	HEMANGIOMA	LYMPHANGIOMA	1

P1–P5= five different pathologists; SL=solar lentigo; LS=lentigo simplex; AK=actinic keratosis; PAK=pigmented AK; LCA=large cell acanthoma; SK=seborrheic keratosis; ISCC=intra-epidermal squamous cell carcinoma; PC=pilar cyst; TC=trichilemmal cyst; PIG=pigmented; BCC=basal cell carcinoma; LENTIG=lentiginous; LPLK=lichen planus like keratosis; JMN=junctional melanocytic naevus

**Table 3:** Pathologists’ Accuracy for Non-Melanocytic Lesion Diagnosis Compared to Majority Diagnosis (BCC, cysts, DF, angioma excluded).

Consensus reference diagnosis	Pathologists’ interpretation		Total interpretation (no)	% Concordance
	Class I	Class V		
Class I BENIGN	**167**	8	175	95%
Class V MALIGNANT	2	**7**	9	78%
Total	169	15	**184**	

**Table 4:** Pathologists’ Accuracy for Non-Melanocytic Lesion Diagnosis Compared to Majority Diagnosis (all cases).

Consensus Reference Diagnosis	Pathologitsts’ Interpretation		Total Interpretation (no)	% Concordance
	Class I	Class V		
Class I BENIGN	**182**	8	190	96%
Class V MALIGNANT	2	**27**	29	93%
Total	184	35	**219**	
